# Immunoproteomics of *Plasmodium falciparum*-infected red blood cell membrane fractions

**DOI:** 10.1590/0074-02760170041

**Published:** 2017-12

**Authors:** Fernanda J Cabral, Luciana G Vianna, Marcia M Medeiros, Bianca Cechetto Carlos, Rosimeire D Martha, Nadia Maria Silva, Luiz Hildebrando P da Silva, Rodrigo G Stabeli, Gerhard Wunderlich

**Affiliations:** 1 Universidade Estadual de Campinas, Instituto de Biologia, Departamento de Biologia Animal, Campinas, SP, Brasil; 2 Universidade de São Paulo, Instituto de Ciências Biomédicas, Departamento de Parasitologia, São Paulo, SP, Brasil; 3 Instituto Butantan, São Paulo, SP, Brasil; 4 Centro de Pesquisa em Medicina Tropical, Porto Velho, RO, Brasil; 5 Fundação Oswaldo Cruz-Fiocruz, Rio de Janeiro, RJ, Brasil; 6 Universidade Nova de Lisboa, Instituto de Higiene e Medicina Tropical, Lisboa, Portugal

**Keywords:** Plasmodium falciparum, symptomatic, asymptomatic, proteomics

## Abstract

**BACKGROUND:**

The surface of infected red blood cells (iRBCs) has been widely investigated because of the molecular complexity and pathogenesis mechanisms involved. Asymptomatic individuals are important in the field because they can perpetuate transmission as natural reservoirs and present a challenge for diagnosing malaria because of their low levels of circulating parasites. Recent studies of iRBC antibody recognition have shown that responses are quantitatively similar in symptomatic and asymptomatic infections, but no studies have characterised the plasmodial proteins targeted by this response.

**OBJECTIVES:**

Our main objective was to identify *Plasmodium falciparum* proteins associated with iRBC ghosts recognised by antibodies in the sera of symptomatic and asymptomatic individuals in the Brazilian Amazon.

**METHODS:**

We collected symptomatic and asymptomatic sera from patients residing in the Brazilian Amazon and *P. falciparum* iRBC ghosts to identify the proteins involved in natural antibody recognition by 2D-electrophoresis, western blotting, and high- resolution mass spectrometry.

**FINDINGS:**

2D gel-based immunoproteome analysis using symptomatic and asymptomatic sera identified 11 proteins with at least one unique peptide, such as chaperones HSP70-1 and HSP70-x, which likely are components of the secretion machinery/PTEX translocon. PfEMP1 is involved in antigenic variation in symptomatic infections and we found putative membrane proteins whose functions are unknown.

**MAIN FINDINGS:**

Our results suggest a potential role of old and new proteins, such as antigenic variation proteins, iRBC remodelling, and membrane proteins, with no assigned functions related to the immune response against *P. falciparum,* providing insights into the pathogenesis, erythrocyte remodelling, and secretion machinery important for alternative diagnosis and/or malaria therapy.

*Plasmodium falciparum* is the causative agent of malaria in tropical areas of the world. The World Health Organization (WHO) recently estimated that approximately 214 million people were infected by *Plasmodium* and 438,000 people died from malaria in 2015 (WHO 2015). Asymptomatic malaria is often characterised by the submicroscopic presence of parasites in the blood of persons with no presence of symptoms. Epidemiological data have revealed the importance of asymptomatic infections in malaria transmission (Alves et al. 2005) and high prevalence of asymptomatic infections in the Brazilian Amazon (Alves et al. 2002, da Silva-Nunes et al. 2012). Although the importance of asymptomatic epidemiology has been well-demonstrated in the Brazilian Amazon, few studies have examined the pathogenesis and antigen recognition in asymptomatic infections. Recently, two studies demonstrated the important aspects of patient serum antibodies in antigen recognition from asymptomatic infections in the Brazilian Amazon. Recombinant expression of merozoite antigens and ultimately recognition of recombinant antigens by symptomatic and asymptomatic plasma antibodies showed that merozoite antigen recognition occurred regardless of symptoms and that other factors may contribute to clinical protection acquisition (Medeiros et al. 2013). Another study correlating the symptomatic/asymptomatic status with infected red blood cell (iRBC) recognition found no striking difference in the frequency and intensity of antibody recognition (Fratus et al. 2014). In contrast, several studies showed that plasmodial proteins displayed on iRBCs are responsible for: (i) targeted antigen recognition and associated immunity and (ii) immune system escape by antigenic variation (Leech et al. 1984, Cheng et al. 1998, Winter et al. 2005, Chan et al. 2012). The well-known variant surface antigens (VSAs), which are related to pathogenesis and antigenic variation, are *P. falciparum* erythrocyte membrane protein 1 (PfEMP1) (Bull et al. 1998), Rifin (repetitive interspersed family) (Kyes et al. 1999), and possibly surface-associated interspersed genes (surfins) (Winter et al. 2005). A recent study showed that PfEMP1 is the main target of naturally acquired antibodies and is associated with protection from age-related clinical manifestations in symptomatic infections (Chan et al. 2012). However, no studies have examined the differences in iRBC recognition by antibodies in (oligo)symptomatic and asymptomatic infections. Additionally, it is unknown which proteins/peptides are the major targets in the recognition by natural antibodies in symptomatic/asymptomatic infections in the Brazilian Amazon. In this study, we applied mass spectrometry analysis using iRBC ghosts, which have infected red blood cell plasma membranes, the erythrocyte's submembrane skeleton, Maurer's clefts, and the protein transport machinery of the parasite to determine which molecules are recognised by symptomatic/asymptomatic serum.

To address the immunproteome, *P. falciparum* patient field isolate 112 (2006) was collected from Rondonia state, Brazil. Parasites were cultured in a candle jar (Jensen & Trager 1977) in RPMI 1640 medium containing 10% human plasma B and B+ erythrocytes. All procedures involving human participants were conducted in accordance with the ethical standards of the Institute of Biomedical Sciences of University of São Paulo research committee (protocol CEPSH 041.11). For assays, parasites were floated in 6% Voluven (Fresenius Kabi, Campinas, Brazil, Lelièvre et al. 2005). After floating for 24-36 h, trophozoites were collected and the erythrocytes were lysed with 0.2x hypotonic solution as described previously (Rabilloud et al. 1999) and iRBC ghosts were stored at −80°C until use. 2D-electrophoresis was conducted in triplicate, and each experiment involved the use of two gels where one was stained with Coomassie blue and the other was transferred onto a membrane and incubated with antibodies. Lysed red blood cell membranes were used as negative controls, and western blotting with symptomatic patient sera (pool of n = 20) and asymptomatic carrier sera (pool of n = 20) revealed no bands (data not shown). Isoelectric focusing was performed as described. Briefly, proteins were homogenised in De-Streak (GE Healthcare, Little Chalfont, UK) buffer and incubated with a 7-cm immobilised pH gradient strip overnight. The following day, the strips were subjected to isoelectric focusing in an ETTAN IPGPHOR (GE Healthcare) apparatus, with a total accumulation of 15,702 Vh for 7 h. For the second dimension, strips were first reduced by 15 min under mild agitation in equilibration buffer (2% SDS, 6 M urea, 75 mM Tris-HCl (pH 8,8) containing 30% glycerol and 0.002% bromophenol blue) and 1% DTT. After reduction, the strips were placed in 0.25% iodoacetamide in the same buffer for 15 min. Electrophoreses were performed in ZOOM 4-20% Tris-glicine gels and in ZOOM 4-20% Bistris gels (Invitrogen, Carlsbad, CA, USA) and stained with Coomassie blue. The resulting gels were maintained in 20% ammonium sulphate until spots were picked. Patient sera were obtained as per the methods described by Medeiros et al. (2013) and Carlos et al. (2016). Western blotting was performed using 1:1000 dilutions of pooled patient sera. The protein spots were recognised, picked from the (NH4)_2_SO_4_-soaked replica gel, reduced, alkylated, and digested overnight with 12.5 ng/μL of trypsin (sequencing grade, Promega, Madison, WI, USA) (Shevchenko et al. 1996) according to the manufacturer's instructions. Extracted peptides were desalted in reversed phase ziptips before liquid chromatography-mass spectrometry (LC- MS/MS) analysis. MS analyses were performed on an LTQ-Orbitrap Velos ETD (Thermo Fisher Scientific): (Thermo Scientific 2012) coupled with an Easy nanoLC II (Thermo Fisher Scientific). The peptides were separated on a C18RP column over a 45-min gradient. Database searching was performed using the SEQUEST algorithm in Proteome discoverer version 1.4 (Thermo Fisher Scientific) against PlasmoDB data (plasmoDB.org) for *P. falciparum* 3D7 and *P. falciparum* IT. The instrument conditions were checked using 50 fmol of a tryptic digest of bovine serum albumin as a standard. Sample carryover was completely removed between runs. All samples were evaluated in duplicate. Sequence analyses of the molecular function, cellular component, and biological processes were conducted using the Gene Ontology (GO) tool on Gene DB databank (www.genedb.org).

We identified five spots in symptomatic sera and six spots in asymptomatic sera (Tables I-II). In Figure (A), a typical result of the recognition profile of the symptomatic sera pool is shown. The identification results are shown in Table I. Analysis of the confidence of the peptides were evaluated based on decoy rates, which represent sequenced peptides with false discovery rates < 1% and confident protein identification with at least a minimum of one unique peptide and one peptide sequence matching (PSM) according to the manufacturer instructions (Thermo Fisher Scientific) (Tables I-II). We identified PfEMP1 (PF3D7_0533100) annotated as truncated var1csa which had one unique peptide and one PSM, reflecting the low abundance of this protein in the sample (Table I). Other proteins were sequenced, such as adenosine deaminase (PF3D7_1029600) with two unique peptides. However, this protein may be a contamination of our ghost preparation with the cellular fraction of the parasite, as this protein has neither an export motif nor any associated function with the iRBC ghost (Table I). Sequencing of another protein (PF3D7_1356500) with 26 PSMs and Gene Ontology (GO) analysis indicated that this protein plays a role in the Golgi network membrane and/or is an integral membrane protein (Table III). Because these peptides were apparently recognised by naturally acquired antibodies, they may be membrane proteins involved in immunity, infection, and/or clinical manifestations.

**Figure:**
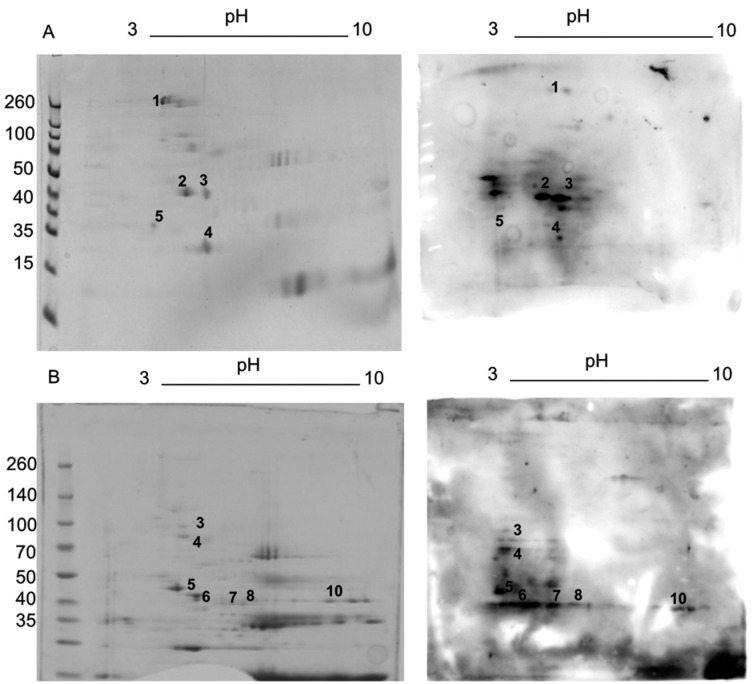
Ghost extracts analysed by 2D-electrophoresis. (A) Right: replica gel of *Plasmodium falciparum* proteins stained with Coomassie blue; left: western blotting with the sera of symptomatic patients. (B) Right: replica gel of *P. falciparum* proteins, stained with Coomassie blue; left: western blotting with the sera of asymptomatic patients from an endemic area.

**TABLE I:** Analysis of sequenced peptides for symptomatic sera

# Spot	Plasmo DB access	Description	Peptides	Unique peptides	PSM	MW(kDa) calculated/observed	pI calculated/observed
1	PF3D7_0533100	organism=Plasmodium_falciparum_3D7 | product=erythrocyte membrane protein 1 (PfEMP1), truncated (VAR1CSA)	KScVMcMQK	1	1	367.7/260	8.37/4.0
2	PfIT_11_v2-1-184488-188775/PF3D7_0611800	organism=Plasmodium_falciparum_3D7 | product=conserved Plasmodium protein, unknown function	LGHKKLSmIR	1	24	12/40	9.82/4.0
3	PF3D7_1029600	organism=Plasmodium_falciparum_3D7 | product=adenosine deaminase, putative	MNLWAVQK VSESQEIDLVK	2	3	42.4/40	5.69/5.0
4	PF3D7_1110900	organism=Plasmodium_falciparum_3D7 | product=ES2 protein, putative	TSLSVVENKKNEK	1	3	69/30	9.22/5.0
5	PfIT_04_v2-3-302028-302201/PF3D7_1356500	organism=Plasmodium_falciparum_3D7 | product=conserved Plasmodium protein, unknown function	IEVTILmcLVK	1	26	7.1/35	9.06/3.0

Proteins and peptides recognised by symptomatic natural antibodies and sequenced by LC-MS/MS. PSM: peptide sequence matching; MW: molecular weight; pI: isoelectric point.

**TABLE II:** Analysis for sequenced peptides for asymptomatic sera

# Spot	Plasmo DB access	Description	Peptides	Unique peptides	PSM	MW (kDa)calculated/observed	pI calculated/observed
3	PF3D7_0818900	organism=Plasmodium_falciparum_3D7 | product=heat shock protein 70 (Hsp70)	TTPSYVAFTDTER	1	5	73.9/100	5.67/4.0
4	PF3D7_0831700	organism=Plasmodium_falciparum_3D7 | product=heat shock protein 70, putative (HSP70-X)	NNLENYcYNVK/ SQVHEIVLVGGSTR/DAGAIAGLNVLR/AmT-KDNNLLGK/NSEN-KEAQNNGPTVEEVN/IY-QGASAQEPQKAEATNLR/IINEPTAAAIAYGLDKK	6	80	75/75	5.77/4.0
4	PF3D7_0818900	organism=Plasmodium_falciparum_3D7 |product=heat shock protein 70 (Hsp70)	LSQDEIDR/ATAG-DTHLGGEDFDNR/NENV-DIIANDQGNR/NENVDII-ANDQGNR/NPENTVFDAK	4	57	73.9/75	5.67/4.0
5	PF3D7_1412500	organism=Plasmodium_falciparum_3D7 | product=actin II (ACT2)	VSPEEHPVLLTEAPLNPK	1	6	17/45	5.15/3.5
5	PF3D7_0818900	organism=Plasmodium_falciparum_3D7 | product=heat shock protein 70 (Hsp70)	TTPSYVAFTDTER	1	5	73.9/45	5.67/3.5
6	PF3D7_1023600	organism=Plasmodium_falciparum_3D7 | product=conserved Plasmodium protein, unknown function	KEKTINNLNK	1	2	25.5/40	9.85/4.5
8	PF3D7_1007800	organism=Plasmodium_falciparum_3D7 | product=conserved Plasmodium protein, unknown function	NDKEEIPDIVNK	1	3	125.2/40	8.5/4.0
10	PF3D7_0918400	organism=Plasmodium_falciparum_3D7 | product=conserved Plasmodium protein	VGTLcTR	1	11	83.3/40	8.48/9.0
10	PF3D7_1369400	organism=Plasmodium_falciparum_3D7 | product=conserved Plasmodium protein, unknown function	LKKNIDLHmQR	1	7	345.2/40	7.34/9.0

Proteins and peptides recognised by asymptomatic natural antibodies and sequenced by LC-MS/MS. PSM: peptide sequence matching; MW: molecular weight; pI: isoelectric point.

**TABLE III:** Gene ontology (GO) for symptomatic sera

Plasmo DB access	Cellular component	Molecular function	Biological process
PF3D7_0533100	Integral component of membrane (GO: 0016021)	Receptor activity (GO: 0004872)	Pathogenesis (GO: 0009405)
PF3D7_0611800	*	*	*
PF3D7_1029600	*	Adenosine deaminase activity (GO: 0004000)	Response to drug (GO: 0042493) Purine ribonuclioside monophosphate biosynthetic process (GO: 0009168)
PF3D7_1110900	*	*	*
PF3D7_1356500	Golgi membrane (GO: 0000139) Integral component of membrane (GO: 0016021)	*	*

Gene ontology (GO) from geneDB databank (www.geneDB.org) for cellular component, molecular function, and biological processes of ghost fraction proteins recognised by serum of symptomatic patients.

* indicates no GO annotation.

The results obtained from the recognition of proteins by sera from asymptomatic persons revealed no striking differences compared with the symptomatic sera recognition profile (Table II). Sera from asymptomatic individuals showed seven spots but it was identified by MS six spots [Figure (B)]. Similarly, some proteins were annotated as proteins with unknown function (PF3D7_0918400 and PF3D7_1369400) and GO analysis showed that these are putative membrane proteins (Table IV). Two other proteins, PF3D7_1023600 and PF3D7_1007800, were identified and are annotated as proteins with unknown function. GO analysis revealed that these proteins are cytoplasmic proteins (Table IV).

**TABLE IV:** Gene ontology (GO), for asymptomatic sera

Plasmo DB access	Cellular component	Molecular function	Biological process
PF3D7_07818900	Cytoplasm (GO:0005737) Nucleus (GO:0005634) Cell surface (GO:0009986)	Protein Binding (GO:0005515) ATPase activity (GO:0016887) Heat shock protein binding (GO:0031072)	Response to unfolded protein (GO:0006986) Response to heat (GO:0009408)
PF3D7_0831700	PTEX-Complex (GO:007619) Symbiont containing vacuole (GO:00200003) Host cell cytoplasm (GO: 0030430) Host cell cytosol (GO:0044164)	Protein binding (GO: 0005515) ATPase activity (GO: 0016887) Heat shock protein binding (GO: 0031072)	*
PF3D7_1023600 unknown	Motor activity (GO: 0003774) ATP_binding (GO: 0005524) Cytoplasm (GO: 0005737)	Cytoplasm (GO: 0005737)	*
PF3D7_1007800 unknown	Cytoplasm	*	*
PF3D7_0918400 unknown	Apicoplast (GO: 002011) Extracellular region (GO: 0005576) Membrane (GO: 0016020) Integral component of membrane (GO: 0016021)	Calcium ion binding (GO: 0005509)	Multicellular organismal development (GO: 007275) Cell differentiation (GO: 0030154)
PF3D7_1369400 unknown	Membrane (GO: 0016020)	*	*

Gene ontology (GO) from geneDB databank (www.geneDB.org) for cellular component, molecular function, and biological processes of ghost fraction proteins recognised by serum of asymptomatic individuals.

* indicates no GO annotation.

We found several hits for heat shock proteins, such as HSP70-1 (PF3D7_0818900) and HSP70-x (PF3D7_0831700), and both were recognised by antibodies from asymptomatic carriers (Tables II-IV). 2D-electrophoresis showed that PfHSP70 and PfHSP70-x have a molecular weight of approximately 75 kDa and pI of 5.5, and were strongly recognised in western blotting (Figure). These data agree with a previous study showing that specific antisera against a PfHSP70-x strongly recognise a 2D-electrophoresis spot of 72.3 kDa with a pI of 5.4 (Grover et al. 2013). Further, GO analysis showed that PfHSP70-x is related to the PTEX complex and is a component of the iRBC cell cytosol (Table IV).

*P. falciparum* iRBC membrane ghosts were previously defined as follow: an iRBC membrane plus Maurer's clefts components, secreted proteins of the parasite to secreted into the cytosol of erythrocytes and erythrocyte submembrane skeleton (Blisnick et al. 2000). We detected proteins related to the membrane, cytoplasm, and erythrocyte cytosol. The predicted secretome of *P. falciparum* indicates that the parasite secretes more than 320 proteins into the erythrocyte cytosol which plays a role in antigenic variation and host erythrocyte remodelling (Hiller et al. 2004, Marti et al. 2004, Maier et al. 2009).

To enable erythrocyte remodelling and presentation of its own antigens on the erythrocyte membrane surface, *P. falciparum* translocates proteins from the parasitophorous vacuole to the erythrocyte membrane via PTEX (Elsworth et al. 2014) translocon, and PfHSP40 and PfHSP70 may be involved in protein assembly after translocation. Functionally, molecular chaperones are important in the remodelling and folding of proteins that are transported into iRBCs (de Koning-Ward et al. 2009, Grover et al. 2013). In this study, we found that PfHSP70 and PfHSP70x are highly recognised by antibodies in asymptomatic sera. In *P. falciparum,* PfHSP70-1 was identified using a proteomic approach as part of Maurer's clefts (Vincensini et al. 2005), although this protein is also highly abundant in the cytosol and parasitophorous vacuole (Pesce & Blatch 2014). Because of its high abundance and importance in protein translocation and serum recognition in our study and others (Vincensini et al. 2005), this protein is a candidate for new drug interventions for malaria treatment to control the parasite in the circulation. Our main finding regarding chaperones is that the HSP70-x sequence contained six unique peptides. Recent studies showed that PfHSP70-x is located in the erythrocyte cytosol and may play a role in trafficking of PfEMP1 to the iRBC surface by forming chaperone complexes/co-complexes within the host erythrocyte (Kulzer et al. 2012). Trafficking of HSP70x is dependent on the PTEX translocon (Rhiel et al. 2016). Our findings for PfHSP70-x suggest that asymptomatic individuals mount an antibody response against chaperone complexes, but the importance of this response remains unclear.

In this study, GO analysis revealed that some proteins are putatively localised on the erythrocyte membrane. Sera from symptomatic patients recognised the protein product of PF3D7_0533100, annotated as PfEMP1 (var1csa). PfEMP1 is well-known as part of the iRBC membrane and is highly associated with malaria pathogenesis and cytoadherence; thus, it is not surprising that symptomatic serum recognised this protein. var1csa expression, based on transcript levels, does not appear to be involved in the allelic exclusion of *var* transcription (Kyes et al. 2003). Additionally, var1csa is annotated as a pseudogene in the 3D7 strain, IT strain, and *P. reichenowi,* and thus the importance of these results is unclear. Here, the field isolate 112 was used as a source of protein, and it is possible that either var1csa is functional in this strain or that other PfEMP1 proteins in this strain contain the peptide sequence. Other prominent and surface exposed antigens such as surfins, rifins, and other PfEMP1s were poorly identified or not identified at all in this study because they were present at very low concentration in our preparations of iRBC ghosts. In addition, when analysing proteins recognised by sera from asymptomatic patient gels, PfEMP1 identification may have failed on account of using bis-Tris gels to resolve proteins by electrophoresis. Another study showed that PfEMP-1 detection by western blotting shows better results when performed using pre-cast Tris-glycine gels or Tris acetate gels (Chan et al. 2012). Even though we successfully identify main virulence antigens, the influence of these antigens on the immune response should not be discarded in both symptomatic and asymptomatic infections.

The humoral immune response against target proteins also depends on the genetic host background and the ability to switch between antibody subclasses. A recent study correlating protein microarray results to immune reactions to IgG and IgM in Africa revealed that several proteins in the parasite are targets of both IgG/IgM responses or uniquely to IgG or IgM. These authors also found that host genetic resistance to malaria is related to the ability to produce immune reactions against several proteins of the parasite (Arama et al. 2015). When we compared proteins recognised by asymptomatic carrier antibodies to those recognised by IgG and IgM in the protein array (Arama et al. 2015), we found that similar proteins (PF3D7_0818900, pF3D7_0831700) were recognised in both the previous study and our study. Another study conducted in the western Amazon using the same sera as used in this study revealed the prevalence of the IgG 1 subclass against surfin proteins in asymptomatic cases, and this tendency was maintained independently of the age of the individual from whom the serum was collected (M Medeiros, unpublished observations). These results indicate that for some individuals, natural resistance to infection and the presence of symptoms arise in early childhood; however, additional studies regarding IgG subclasses and IgM in relation to the protection from symptoms of malaria should be conducted in the Brazilian Amazon to confirm this hypothesis.

The main finding of this study is that proteins related to iRBC ghosts may be important factors in antibody responses and are correlated to the presence and absence of malaria symptoms. The identified antigens may influence the outcome of malaria and are potential targets of future studies on their function and localisation.
